# On correlation between canopy vegetation and growth indexes of maize varieties with different nitrogen efficiencies

**DOI:** 10.1515/biol-2022-0566

**Published:** 2023-03-23

**Authors:** Xia Zhao, ShuaiLi Wang, Tao Wen, Jiamin Xu, Bao Huang, Shufeng Yan, Gangqiang Gao, Yali Zhao, Hongping Li, Jiangfang Qiao, Jinliang Yang, Lianhai Wu, Hongwei Wang, Tianxue Liu, Xinyuan Mu

**Affiliations:** Cereal Institute, Henan Academy of Agricultural Sciences/Henan International Joint Laboratory on Maize Precision Production, Zhengzhou 450002, Henan, China; College of Agronomy, Henan Agricultural University, Zhengzhou 450002, China; Department of Agronomy and Horticulture, University of Nebraska-Lincoln, Lincoln, NE 68583-0915, USA; Sustainable Soils and Grassland Systems Department, Rothamsted Research, North Wyke, Okehampton EX20 2SB, UK; Wancheng District Public Complaints and Proposals Bureau, Henan, Nanyang 473000, China

**Keywords:** maize, nitrogen efficiency, vegetation indices, leaf nitrogen content, dry matter content

## Abstract

Studying the canopy spectral reflection characteristics of different N-efficient maize varieties and analyzing the relationship between their growth indicators and spectral vegetation indices can help the breeding and application of N-efficient maize varieties. To achieve the optimal management of N fertilizer resources, developing N-efficient maize varieties is necessary. In this research, maize varieties, i.e., the low-N-efficient (Zhengdan 958, ZD958), the high-N efficient (Xianyu 335, XY335), the double-high varieties (Qiule 368, QL368), and the double inefficient-type varieties (Yudan 606 YD606), were used as materials. Results indicate that nitrogen fertilization significantly increased the vegetation indices NDVI, GNDVI, GOSAVI, and RVI of maize varieties with different nitrogen efficiencies. These findings were consistent with the performance of yield, dry matter mass, and leaf nitrogen content and were also found highest under both medium and high nitrogen conditions in the double-high variety QL368. The correlations of dry matter quality, leaf nitrogen content, yield, and vegetation indices (NDVI, GNDVI, RVI, and GOSAVI) at the filling stage of different N-efficient maize varieties were all highly significant and positive. In this relationship, the best effect was found at the filling stages, with correlation coefficients reaching 0.772–0.942, 0.774–0.970, 0754–0.960, and 0.800–0.960. The results showed that the yield, dry matter weight, and leaf nitrogen content of maize varieties with different nitrogen efficiencies increased first and then stabilized with the increase in the nitrogen application level in different periods, and the highest nitrogen application level of maize yield should be between 270 and 360 kg/hm^2^. At the filling stage, canopy vegetation index of maize varieties with different nitrogen efficiencies was positively correlated with yield, dry matter weight, and leaf nitrogen content, especially GNDVI and GOSAVI on the leaf nitrogen content. It can be used as a means to predict its growth index.

## 1 Introduction

Among the essential nutrients of plants, nitrogen is the primary chemical element that limits the biological production and yield. So nitrogen fertilizer plays an important role in crop growth. Its abundance and deficiency can affect crop metabolism, physiological characteristics, and nutrient absorption and utilization directly [1]. There were significant differences in nitrogen absorption and utilization between different N-efficient varieties of maize crop [2,3]. According to the nitrogen use efficiency (NUE) of different maize varieties, it can be divided into four types: higher grain yield under high nitrogen and low nitrogen levels, higher grain yield only under low nitrogen level, higher grain yield only under high nitrogen level, and lower than average grain yield under high nitrogen and low nitrogen levels [4,5], namely, double efficient type, low nitrogen efficient type, high nitrogen efficient type, and double inefficient type. Accurate and timely evaluation of crop growth and development as well as leaf analysis is the basis for reasonable fertilization, and it is also necessary to monitor the growth status of crops, along with [6]. Studies have shown that in wheat, rice, and other crops, leaf area, biomass, and leaf color are used to characterize the growth of crops [7,8,9]. A number of results have been achieved in the use of spectral imaging for crop growth and nutrient monitoring. The optimal spectral variables of key growth periods were used as parameters to model the N nutrition of winter wheat [7,8,9]. When using multispectral and spectral data to invert the adaptability of the maize leaf area index, it was found that the vegetation index could accurately reflect the maize leaf area index in the study area [10,11]. The results show that the spectral vegetation index (GNDVI, PSSRc, NDVI4, and DI) has higher fitting effects with the amount of aboveground dry matter in maize at different growth stages [12]. The optimal spectral vegetation index for summer maize LAC estimation at different growth stages had good results [13]. However, in the process of agricultural production, due to the complexity of the background factors for maize growth, the use of spectral vegetation index to monitor the growth status of maize varieties with different nitrogen efficiencies is rarely studied. Especially for maize hybrids with significant differences in genetic background, the single spectral vegetation index is difficult to reflect the differences in nitrogen absorption and utilization [14]. In this study, the relationship between yield, biomass, and leaf nitrogen content under different nitrogen levels was demonstrated, which provided a possibility for the optimal management of nitrogen resources in the production of maize varieties with different nitrogen efficiencies. Through the correlation between canopy vegetation index and growth index, the possibility of UAV spectroscopy to predict maize varieties and growth indexes under different nitrogen efficiencies was explored.

The trials for the present experiment were conducted in 2021 in Yuzhou, Xuchang, Henan (113°56′E, 34°09′N), China. The test sites were flat with uniform ground force. In the individual test field, the drainage and irrigation were convenient, and the planting system was two ripe wheat-maize rotations per year. The soil pH was 7.5 and the organic matter content was 24.8 g/kg. The alkaline nitrogen and the available potassium content were 98.34 and 152.22 mg/kg, respectively. While the content of available phosphorus was 30.76 mg/kg. In this experiment, four representative maize varieties with different nitrogen efficiencies were selected as the research objects. the low-N efficient varieties (Zhengdan 958, ZD958), the high-N efficient varieties (Xianyu 335, XY335), the efficient-efficient varieties (Qiule 368, QL368), and the nonefficient-nonefficient varieties (Yudan 606, YD606) were used as test materials [5,15].

### 1.1 Field trial design and management

A split-plot experimental design was used in this study. The five nitrogen application levels in the main zone are 0, 90, 180, 270 and 360 kg/hm^2^, namely N0, N90, N180, N270, and N360, respectively, for 0, 90, 180, 270, and 360 kg/hm^2^ N application rates. Four different nitrogen efficiency maize varieties in the sub-zone are the low-N-efficient (Zhengdan 958, ZD958), the high-N efficient (Xianyu 335, XY335), the double-high varieties (Qiule 368, QL368) and the double inefficient-type varieties (Yudan 606 YD606). The experimental planting density was 67,500 plants/hm^2^ with three replicates and for a total of 60 plots. The area of each plot was 6.0 m × 3.6 m (6 rows) in size. The amount of potassium and phosphorus fertilizer applied to each treatment was 150 kg/hm^2^. All fertilizers were applied at one time during the jointing period by artificial inter-row trenching. The trial was sown on June 15, 2021, and harvested on September 29, 2021, with other field management being the same as local fields (from Henan Xinlianxin Chemical Industry Group Co).

### 1.2 Ground-truth data collection

Twenty representative plants with uniform growth were selected from each plot at the jointing stage for listing, and then, three representative above-ground parts of maize plants were selected from each plot at the trumpet stage, the filling stage, and the maturity stage, including stem, sheath, leaf, bract, stem axis, and grain. After sampling, the samples were divided into two parts: stem sheath and leaf at the large bell-mouth stage; into three parts: stem sheath (stem and leaf sheath), leaf blade, and ear at the filling stage; and into four parts: stem sheath (stem and leaf sheath), leaf blade, bract shaft (bract and cob shaft), and grain at the maturity stage. The samples were packed into kraft paper bags. Then, they were put in the oven (Yamato-dec912c, made in Japan) for 30 min at 105°C, dried at 80°C to constant weight, cooled to room temperature, and weighed with a one-tenth balance (Mettler Toledo-me303e, made in China). The weighed samples were crushed by Taiste-fw100, made in China, and then, 0.1 g of the crushed and screened samples was weighed in a deboiling tube, with 5 mL of concentrated sulfuric acid for deboiling, H_2_O_2_ as catalyst, in a continuous flow analyser (AA3, SEAL-Analytical System, made in Germany) to determine the plant and grain nitrogen content [16]. In the mature stage of maize, the middle 2 rows of each plot were selected to harvest all the ears, and 10 representative ears were selected for the determination of ear length, ear diameter, row number of ears, row number of grains, 100 grain weight, grain number per ear, and grain weight per ear. Then, all the ears were threshed and the grain yield was converted according to 14% water. The data from this part were used to calculate the indicators related to dry matter and nitrogen accumulation.

### 1.3 The UAV platform, image acquisition and processing

**Figure 1:**
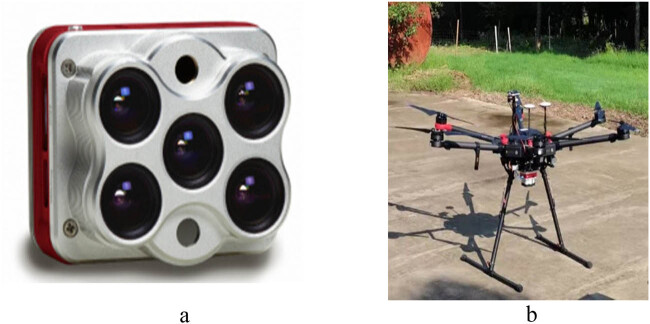
(a) A MicaSense Rededge-Altum. (b) A DJI Matrice 600 Pro UAV platform (SZ DJI Technology Co., Shenzhen, China).

The multispectral remote sensing system of the UAV was used to obtain spectral information. The system consists of a six-rotor drone, a DJI Matrice 600 Pro UAV (SZ DJI Technology Co., Shenzhen, China) (Figure 1b). This system is equipped with a MicaSense Rededge-Altum (Figure 1a). The MicaSense Rededge-Altum did collect five discontinuous spectral bands of red, green, blue, red edge, near infrared, and thermal infrared with pixel size of 3.45 μm, resolution of 2,064 × 1,544, and field of view of 48 × 36.8°.

In the large trumpet stage, grouting period, and mature stage, the UAV multispectral remote sensing system was used to carry out information collection. For the purpose, clear, windless weather, and a working time between 10:00 and 14:00 h were chosen. Before the flight of the drone, the route was demarcated, the gray board was used for correction, and the aircraft flight direction was set east–west, with 80% for both the heading overlap and side overlap. The acquired multispectral photos were imported into Pi×4D software in sequence for image processing and the stitched images were corrected geometrically with high-definition digital images. Finally, the band operation was performed using ENVI 5.3 to extract the parameters. Microsoft Excel was used for data sorting and SPSS 19.0 software was used for statistical analysis, inter-processing significance test (Duncan’s), and correlation.

## 2 Results

### 2.1 Effects of nitrogen supply levels on yield

It can be seen from Table 1 that the yield of maize varieties with different nitrogen efficiencies increases first with the increase in nitrogen application and then tends to be stable. Under the conditions of N0, N90, N180, N270, and N360, the coefficients of variation between grain yield varieties were 24.94, 9.37, 11.29, 11.44, and 12.91%, respectively, and the rate of nitrogen application increased first and then decreased. Under the same nitrogen fertilizer conditions, except for N90, there were significant differences in grain yield between varieties, and the yield of YD606 was the lowest. And under medium and high nitrogen conditions, the yield of QL368 was the highest. Under the treatment of N0, N90, N270, and N360, the grain yield of ZD958 was 83.15, 15.89, 11.34, and 16.83% higher, respectively, than that of YD606, the grain yield of XY335 was 28.55, 0.19, 1.33, and 7.40% higher than that of YD606, and the grain yield of QL368 was 46.70, 19.29, 27.26, and 34.26% higher than that of YD606.

**Table 1:** Effects of nitrogen supply level on yield of maize varieties with different nitrogen efficiencies

	ZD958	XY335	YD606	QL368
N0	8721a	8327a	7220b	8887a
N90	9431a	9964a	8866a	10362a
N180	10153b	10364ab	10065b	10889a
N270	11521a	11974a	10724b	11916a
N360	11454a	11772a	10480b	11717a
Nitrogenous fertilizer (F)	61.84*			
Cultivars (C)	19.12*			

Note: Different lowercase letters of different varieties indicate significant differences among varieties (*P* < 0.05). “*” indicates the significance at the 0.01 level.

### 2.2 Effects of nitrogen supply levels on the leaf nitrogen content

It can be seen that the effect of nitrogen fertilizer treatment on the leaf nitrogen content of maize at different growth stages reached a very significant level, and there were significant differences in the leaf nitrogen content of maize cultivars with different nitrogen efficiencies at the filling stage (Table 2). Under the conditions of N0, N90, N180, N270, and N360, the coefficients of variation between cultivars with the leaf nitrogen content at different growth stages were 21.63, 9.74, 5.91, 17.11, 7.10, 26.21, 13.66, 13.75, 8.62, 10.54, 18.71, 8.63, 10.70, 17.19, and 5.88%, respectively, which decreased first and then increased with the increase in nitrogen application. With the increase in nitrogen application, the leaf nitrogen content of maize varieties with different nitrogen efficiencies increased first and then was stable. Under the condition of no nitrogen application, there was no significant difference in the leaf nitrogen content of different N-efficient maize cultivars at the filling stage and the mature stage. Under the same nitrogen fertilizer condition, YD606 had the lowest nitrogen content in leaves at both the trumpet stage and the filling stage. Under high nitrogen conditions, the leaf nitrogen content of QL368 at the silking stage was significantly higher than that of ZD958, XY335, and YD606.

**Table 2:** Effects of nitrogen supply level on the leaf nitrogen content of maize varieties with different nitrogen efficiencies

N level	Cultivar	Trumpet stage (kg/hm^2^)	Filling stage (kg/hm^2^)	Maturity (kg/hm^2^)
N0	ZD958	24.81bc	27.15a	17.97a
	XY335	33.31a	24.92a	12.87a
	YD606	19.81c	14.70b	11.89a
	QL368	29.12ab	28.44a	14.26a
	F	7.02*	7.05*	1.78 ns
N90	ZD958	48.56a	44.64b	27.85a
	XY335	53.57a	57.41a	23.63a
	YD606	43.29a	43.87b	25.88a
	QL368	44.58a	44.71b	23.15a
	F	1.45 ns	6.70*	1.00 ns
N180	ZD958	64.31a	65.61ab	29.30a
	XY335	56.67a	58.05bc	23.79a
	YD606	57.55a	50.93c	30.11a
	QL368	61.43a	69.99a	26.10a
	F	1.13 ns	6.94*	0.75 ns
N270	ZD958	57.21a	62.67a	35.57a
	XY335	62.41a	67.74a	26.27bc
	YD606	69.11a	59.95a	29.90b
	QL368	84.12a	72.78a	24.22c
	F	1.40 ns	1.20 ns	9.92**
N360	ZD958	60.20a	65.22a	29.47a
	XY335	71.09a	66.64a	33.47a
	YD606	66.25a	56.25a	31.18a
	QL368	69.06a	72.93a	29.79a
	F	1.65 ns	1.70 ns	1.50 ns
ANOVA	C	1.88 ns	9.56**	2.34 ns
	F	41.69**	82.11**	19.23**
	C × F	1.52 ns	1.38 ns	0.77 ns

Note: Different lowercase letters of different varieties indicate significant differences among varieties (*P* < 0.05). “*” indicates the significance at the 0.05 level. “**” indicates the significance at the 0.01 level.

### 2.3 Effects of nitrogen supply levels on dry matter accumulation

It can be seen that the effect of nitrogen fertilizer treatment on the dry matter accumulation of maize at different growth stages reached a highly significant level, and the influence of varieties on the dry matter accumulation of maize at the filling stage and the maturity stage also reached a highly significant level (Table 3). Under the conditions of N0, N90, N180, N270, and N360, the coefficients of variation between biomass cultivars at different growth stages were 17.89, 2.80, 7.88, 7.01, 11.33, 23.00, 11.79, 13.09, 5.06, 7.95, 24.25, 7.10, 13.46, 7.43, and 14.43%. Under the condition of N0, the differences among varieties at different growth stages were the greatest. With the increase in nitrogen application, the dry matter accumulation of maize varieties with different nitrogen efficiencies increased first and then decreased. Under the same nitrogen application condition, YD606 had the lowest dry matter accumulation at different growth stages, and QL368 had the highest dry matter accumulation at the filling stage and the maturity stage.

**Table 3:** Effects of nitrogen supply level on dry matter content of maize varieties with different nitrogen efficiencies

N level	Cultivar	Trumpet stage (kg/hm^2^)	Filling stage (kg/hm^2^)	Maturity (kg/hm^2^)
N0	ZD958	3445.66a	6814.35b	9867.43ab
	XY335	2811.26a	6567.04b	8598.79bc
	YD606	2329.08a	4565.73c	6476.94c
	QL368	3426.53a	8218.65a	11778.76a
	F	2.22 ns	16.94**	6.64*
N90	ZD958	3671.97a	10370.38ab	13515.83a
	XY335	3732.19b	12207.77a	14867.34a
	YD606	3507.45a	9702.00ab	14012.74a
	QL368	3565.53a	9504.18b	15872.52a
	F	0.88 ns	2.73 ns	0.67 ns
N180	ZD958	4202.19a	11171.13ab	15204.54b
	XY335	4252.36a	11014.22ab	13577.34b
	YD606	3587.39a	9322.75b	15548.42b
	QL368	3851.00a	12878.27a	18649.97a
	F	1.10 ns	3.32 ns	6.164*
N270	ZD958	4964.10a	11497.98a	16391.80a
	XY335	4348.66a	12263.84a	17278.87a
	YD606	4702.03a	11661.05a	15363.65a
	QL368	4275.75a	12835.29a	18297.20a
	F	0.84 ns	0.31 ns	0.88 ns
N360	ZD958	4545.51ab	11026.16a	14814.16a
	XY335	3991.02b	12317.56a	17667.53a
	YD606	4431.75b	11703.94a	14246.56a
	QL368	5236.37a	13288.74a	19278.06a
	F	4.93*	1.46 ns	2.38 ns
ANOVA	C	2.17 ns	5.75**	8.45**
	F	17.16**	33.21**	26.90**
	C × F	1.33 ns	1.41 ns	0.95 ns

Note: Different lowercase letters of different varieties indicate significant differences among varieties (*P* < 0.05). “*” indicates the significance at the 0.05 level. “**” indicates the significance at the 0.01 level.

### 2.4 The normalized vegetation index (NDVI)

**Figure 2:**
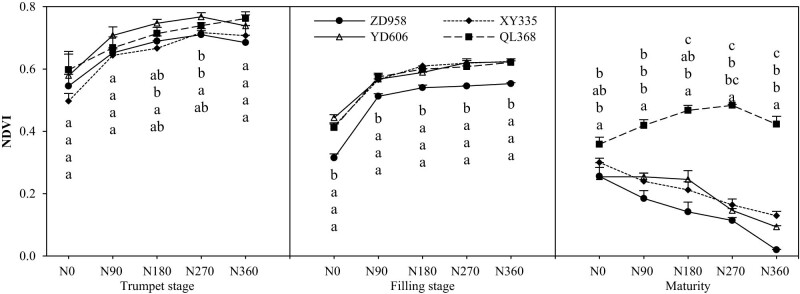
Effects of nitrogen supply level on canopy vegetation index (NDVI) of maize varieties with different nitrogen efficiencies. Note: Different lowercase letters of different varieties indicate significant differences among varieties (*P* < 0.05). The same as below.

The NDVI of maize varieties with different nitrogen efficiencies decreased with the growth process, and the NDVI decreased more in the later growth period than in the early growth period (Figure 2). Under the conditions of N0, N90, N180, N270, and N360, the coefficients of variation of biomass between varieties of maize cultivars with different nitrogen efficiencies were 7.95, 4.21, 4.88, 3.49, 4.72, 14.18, 5.19, 5.30, 5.6, 5.78, 16.70, 36.81, 52.66, 75.92, and 106.64%, respectively. In addition to the maturity period, the coefficient of variation between varieties was largest under the condition of N0 and then showed a decreasing trend and finally an upward trend. Under the same nitrogen application conditions, the low-N efficient cultivar (ZD958) was the lowest. In addition to the maturity stage, the canopy vegetation index (NDVI) of maize cultivars with different nitrogen efficiencies increased first and then stabilized with the increase of nitrogen application. In addition to QL368 at the maturity stage, the maize canopy vegetation index NDVI decreased with the increase in nitrogen application.

### 2.5 The green-normalized vegetation index (GNDVI)

**Figure 3:**
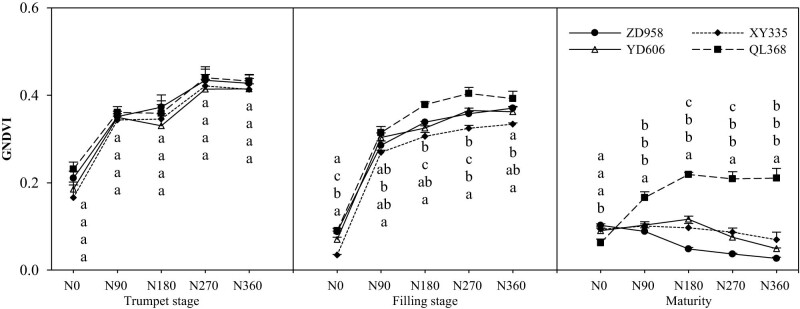
Effects of nitrogen supply level on canopy vegetation index (GNDVI) of maize varieties with different nitrogen efficiencies.

It can be seen that nitrogen treatments and varieties have significant effects on the GNDVI of maize varieties with different nitrogen efficiencies (Figure 3). With the progress of growth, the GNDVI of maize canopy vegetation index showed a decreasing trend, and the decreasing range was larger in the late growth period. Under the conditions of N0, N90, N180, N270, and N360, the variation coefficients of maize varieties with different nitrogen efficiencies at different growth stages were 14.39, 2.07, 5.20, and 2.71%; 2.26, 36.14, 6.71, 9.14, 9.04, and 6.62%; and 19.81, 30.43, 59.55, 72.80, and 93.03%. The GNDVI of maize canopy vegetation index increased significantly with increasing N fertilizer application compared with no N fertilizer. With the increase in the nitrogen application rate, GNDVI increased first and then stabilized with the increase in the nitrogen application rate except at the maturity stage. Under medium and high nitrogen conditions, the GNDVI of the efficient-efficient cultivar (QL368) at all growth stages was significantly higher than that of ZD958, XY335, and YD606. Under high nitrogen conditions, the GNDVI of QL368 was the highest. Except for the N0 treatment, the GNDVI of the low-N efficient cultivar (ZD958) was lower than that of other varieties at the maturity stage, and the GNDVI of QL368 was the highest. However, there was no significant difference between XY335 and YD606.

### 2.6 The green-optimized soil conditioning vegetation index (GOSAVI)

**Figure 4:**
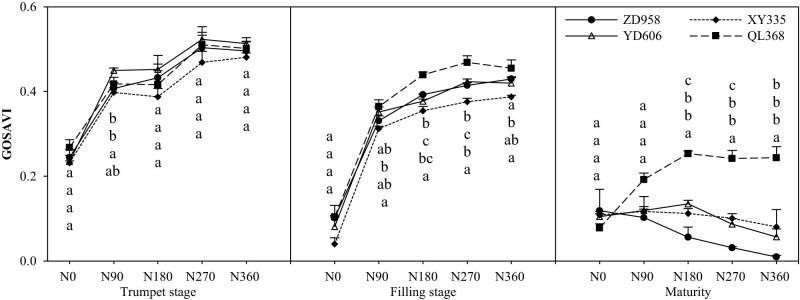
Effects of nitrogen supply level on canopy vegetation index (GOSAVI) of maize varieties with different nitrogen efficiencies.

The effect of nitrogen supply levels on the GOSAVI of different nitrogen efficiency maize varieties reached a significant level (Figure 4). With the growth period, the GOSAVI of different nitrogen efficiency maize varieties decreased, and the lower rate was greater in the late growth period. Except for the mature stage, the coefficient of variation among varieties was the largest under the condition of N0, which increased first and then decreased with the increase in the nitrogen application rate. With the increase in the nitrogen application rate, GOSAVI of maize varieties with different nitrogen efficiencies increased continuously and then tended to be stable. At maturity, GOSAVI of ZD958, XY335, and YD606 decreased with the increase in the nitrogen application rate. Under medium and high nitrogen conditions, the GOSAVI of QL368 was significantly higher than that of other cultivars at different growth stages. Under high nitrogen condition, GOSAVI of the high-N efficient varieties XY335 was significantly lower than that of other varieties during the filling stage, and there was no significant difference between ZD958 and YD606.

### 2.7 The ratio of vegetation index (RVI)

**Figure 5:**
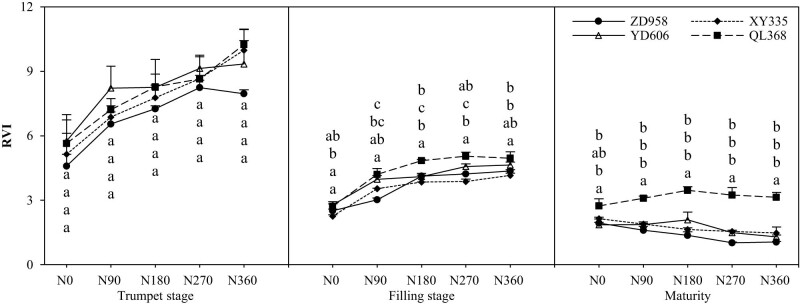
Effects of nitrogen supply level on canopy vegetation index (RVI) of maize varieties with different nitrogen efficiencies.

The effect of nitrogen supply levels on the RVI of maize varieties with different nitrogen efficiencies reached a significant level (Figure 5). During the growth process, the RVI of different nitrogen efficiency maize varieties decreased continuously, and the decline in the late growth period of maize was smaller than that in the early growth stage. In addition to the maturity period, the coefficient of variation of maize varieties decreased first and then increased with the increase in nitrogen application. The RVI of maize varieties with different N efficiencies increased with the increase in the N application rate at the big trumpet stage and the filling stage. At the maturity stage, the RVI of maize decreased with the increase in nitrogen application, except for the efficient varieties QL368. At the filling stage and the maturity stage, the RVI of QL368 was significantly higher than that of ZD958, XY335, and YD606 under medium and high nitrogen conditions, while the RVI of XY335 was significantly lower than that of other varieties. There was no significant difference in RVI between ZD958 and YD606.

### 2.8 Relationships between canopy vegetation index and yield

There was a significant positive correlation between canopy vegetation index and maize yield in the trumpet opening stage and the filling stage, and the correlation was significantly higher than that in the mature stage (Table 4). There was a significant positive correlation between the variety mixing at the bell-mouth stage and the filling stage, but the correlation coefficient was lower than that of single variety. In the trumpet stage, the correlation coefficient between NDVI and yield was 0.748–0.827. The correlation coefficient between GNDVI and yield was 0.809–0.895. The correlation coefficient between RVI and yield was 0.567–0.840, and that between GOSAVI and yield was 0.848–0.937. In the filling stage, the correlation coefficient between NDVI and yield was 0.775–0.963, that between GNDVI and yield was 0.809–0.970, that between RVI and yield was 0.754–0.927, and that between GOSAVI and yield was 0.809–0.960 (Table 4).

**Table 4:** Correlation between canopy vegetation index NDVI, GNDVI, RVI, GOSAVI, and yield at different growth stages

Stage	Cultivar	NDVI	GNDVI	RVI	GOSAVI
Trumpet stage	ZD958	0.823**	0.809**	0.796**	0.848**
	XY335	0.827**	0.835**	0.840**	0.886**
	YD606	0.748**	0.895**	0.564*	0.937**
	QL368	0.777**	0.857**	0.786**	0.857**
	Mixture	0.681**	0.810**	0.617**	0.794**
Filling stage	ZD958	0.775**	0.809**	0.754**	0.809**
	XY335	0.829**	0.844**	0.826**	0.839**
	YD606	0.963**	0.970**	0.927**	0.960**
	QL368	0.917**	0.904**	0.877**	0.897**
	Mixture	0.655**	0.853**	0.787**	0.848**
Maturity stage	ZD958	−0.806**	−0.813**	−0.823**	−0.658**
	XY335	−0.763**	−0.763**	−0.763**	−0.763**
	YD606	−0.512	−0.177	−0.223	−0.182
	QL368	0.623*	0.836**	0.376	0.833**
	Mixture	0.014	0.299*	0.090	0.236

“*” indicates the significance at the 0.05 level. “**” indicates the significance at the 0.01 level.

### 2.9 Relationship between canopy vegetation index and leaf nitrogen content

There was a significant positive correlation between canopy vegetation index and leaf nitrogen content at the filling stage, followed by the big trumpet stage, and the lowest correlation at the maturity stage (Table 5). In the big trumpet stage and the filling stage, the correlation leaf of variety mixing showed extremely significant positive correlation, and the correlation coefficient was lower than that of single variety. At the filling stage, the correlation coefficient between NDVI and leaf nitrogen content was 0.839–0.979, that between GNDVI and leaf nitrogen content was 0.871–0.958, and that between RVI and leaf nitrogen content was 0.851–0.960. The correlation coefficient between GOSAVI and leaf nitrogen content was 0.873–0.957.

**Table 5:** Correlation between canopy vegetation index NDVI, GNDVI, RVI, GOSAVI, and leaf nitrogen content in maize at different growth stages

Stage	Cultivar	NDVI	GNDVI	RVI	GOSAVI
Trumpet stage	ZD958	0.827**	0.808**	0.814**	0.826**
	XY335	0.820**	0.871**	0.653**	0.860**
	YD606	0.647**	0.882**	0.551*	0.918**
	QL368	0.513	0.704**	0.532*	0.704**
	Mixture	0.611**	0.770**	0.580**	0.775**
Filling stage	ZD958	0.908**	0.939**	0.960**	0.939**
	XY335	0.853**	0.877**	0.851**	0.874**
	YD606	0.979**	0.958**	0.931**	0.957**
	QL368	0.839**	0.871**	0.909**	0.873**
	Mixture	0.777**	0.869**	0.817**	0.869**
Maturity	ZD958	0.779**	0.777**	0.746**	0.812**
	XY335	0.617*	0.733**	0.650**	0.733**
	YD606	0.567*	0.761**	0.386	0.876**
	QL368	0.418	0.622*	0.301	0.613*
	Mixture	0.124	0.314*	0.160	0.297*

“*” indicates the significance at the 0.05 level. “**” indicates the significance at the 0.01 level.

### 2.10 Relationship between canopy vegetation index and biomass

There was a significant positive correlation between canopy vegetation index and maize yield at the grain-filling stage, and the correlation was significantly higher than that at the trumpet stage and the maturity stage (Table 6). At the filling stage, the correlation of variety mixing was significantly positive, but the correlation coefficient was lower than that of single variety. At the filling stage, the correlation coefficient between NDVI and dry matter quality was 0.772–0.942. The correlation coefficient between GNDVI and dry matter quality was 0.774–0.927. The correlation coefficient between RVI and dry matter mass was 0.787–0.878, and that between GOSAVI and dry matter mass was 0.777–0.927.

**Table 6:** Correlation between maize canopy vegetation index NDVI, GNDVI, RVI, GOSAVI, and dry matter quality in different growth stages

Stage	Cultivar	NDVI	GNDVI	RVI	GOSAVI
Trumpet stage	ZD958	0.843**	0.823**	0.806**	0.847**
	XY335	0.774**	0.802**	0.787**	0.800**
	YD606	0.907**	0.882**	0.878**	0.884**
	QL368	0.404	0.736**	0.462	0.736**
	Mixture	0.546**	0.675**	0.500**	0.666**
Filling stage	ZD958	0.942**	0.927**	0.828**	0.927**
	XY335	0.774**	0.802**	0.787**	0.800**
	YD606	0.907**	0.882**	0.878**	0.884**
	QL368	0.772**	0.774**	0.854**	0.770**
	Mixture	0.742**	0.793**	0.743**	0.792**
Maturity	ZD958	−0.516*	−0.705**	−0.702**	−0.378
	XY335	−0.681**	−0.328	−0.681**	−0.280
	YD606	−0.397	−0.055	−0.172	−0.062
	QL368	0.481	0.662**	0.203	0.654**
	Mixture	0.124	0.314*	0.161	0.297*

“*” indicates the significance at the 0.05 level. “**” indicates the significance at the 0.01 level.

## 3 Discussion

With the increase in nitrogen application, the yield, dry matter accumulation, and nitrogen accumulation of maize showed a trend of first increasing and then stabilizing, and the high-efficiency varieties were significantly higher than the nitrogen-inefficient varieties [17]. This conclusion is similar to the results of this study. Previous studies showed that both nitrogen application level and variety affected crop canopy reflectance [18,19]. The application of nitrogen fertilizer increased the near-infrared band reflectance of the rice canopy and reduced the reflectivity of the visible light band [20,21]. Increasing N fertilizer application could increase NDVI, GNDVI, RVI, and GOSAVI of rice (Table 7). Fu et al. also found that leaf N content of rice was positively correlated with canopy vegetation indices ndvi, rvi, dvi, and gndvi [22]. The results of this study showed that the differences of dry matter weight and leaf nitrogen concentration of maize varieties with different nitrogen efficiencies at different nitrogen application levels were consistent with their own variety characteristics. With the increase in the nitrogen application level, the yield, dry matter weight, and leaf nitrogen content of maize varieties with different nitrogen efficiencies increased first and then stabilized. The canopy vegetation index (NDVI, GNDVI, GOSAVI, RVI) and growth index of maize varieties at different stages and with different nitrogen efficiencies were significantly different, and the canopy vegetation index (NDVI, GNDVI, GOSAVI, RVI) and growth index increased with the increase in the nitrogen application rate, which indicated the possibility of canopy vegetation index predicting growth index. Ryckewaert et al. analyzed the spectral data of different genotypes of maize varieties under water stress and screened out the more drought-tolerant maize varieties [23]. Fan et al. showed that nitrogen application rate had different effects on canopy vegetation index of different maize varieties [24]. The results showed that there was no significant difference in canopy vegetation index of maize varieties with different N efficiencies under low N conditions. With the increase in the nitrogen application rate, the difference of canopy vegetation index of maize varieties with different nitrogen efficiencies increased. QL368 had the highest canopy vegetation indices (NDVI, GNDVI, RVI, and GOSAVI) under medium and high nitrogen conditions, and its yield, dry matter weight, and leaf nitrogen content were also higher than those of ZD958, XY335, and YD606. The results indicated that maize varieties with high nitrogen efficiency had strong greenness, higher contents of chlorophyll A, chlorophyll B, and carotenoid in leaves, and higher absorption rates of visible light, especially red, blue, and violet light, resulting in higher canopy vegetation indices NDVI, GNDVI, RVI, and GOSAVI, and higher response to nitrogen supply, strong material production capacity, and higher yield [25,26]. This indicates that index does not have the ability to screen high-efficiency maize varieties under low nitrogen condition and canopy vegetation, while canopy vegetation index provides the possibility to screen high-efficiency maize varieties under non-low nitrogen condition. There is a correlation between canopy vegetation index and crop yield, dry matter quality, and leaf nitrogen content. Phyu et al. found that NDVI was significantly associated with rice yields [27]. Niu et al. found that the vegetation indices derived from UAV RGB imagery had great potential to estimate maize above-ground biomass with *R* ^2^ values ranging from 0.63 to 0.73. When estimating the above-ground biomass of maize by using multivariable linear regression based on vegetation indices, a higher correlation was obtained with an *R* ^2^ value of 0.82 [28]. Panek et al. found that early spring NDVI in Croatia, the Czech Republic, Germany, Hungary, Latvia, Lithuania, Poland, and Slovakia was very closely related to cereal yields [29]. The results of this study showed that maize yield, dry matter mass, and leaf nitrogen content had similar trends with canopy vegetation indices (NDVI, GNDVI, RVI, and GOSAVI) among different nitrogen levels or maize varieties. Therefore, this experiment analyzed the correlation between yield (Table 4), dry matter mass (Table 6), leaf nitrogen content (Table 5), and canopy vegetation index at the trumpet stage, the filling stage, and the maturity stage. The results showed that maize yield, dry matter mass, and leaf nitrogen content were significantly positively correlated with canopy vegetation index at the filling stage, and the correlation coefficient was higher than that at the trumpet stage and the mature stage. The correlation coefficient between leaf nitrogen content and four canopy vegetation indices was higher than that of yield and dry matter mass at the filling stage (Table 5). However, the correlation between GNDVI and GOSAVI and leaf nitrogen content, yield, and dry matter quality in the canopy vegetation index at the filling stage was stronger than that of other vegetation indexes. Therefore, it provided the possibility for the canopy vegetation index of maize varieties with different nitrogen efficiencies to predict the leaf nitrogen content, yield, and dry matter quality at the filling stage, and the prediction of the leaf nitrogen content might be more accurate.

**Table 7:** Vegetation indices involved in this study

Vegetation index	Formulas	Reference
NDVI	NDVI = (NIR − R)/(NIR + R)	[21]
GNDVI	GNDVI = (NIR − G)/(NIR + G)	[11]
RVI	RVI = NIR/RED	[30]
GOSAVI	GOSAVI = (1 + 0.16) × (NIR − G)/(NIR + G)	[24]

## 4 Conclusion

The results showed that the yield, dry matter weight, and leaf nitrogen content of maize varieties with different nitrogen efficiencies increased first and then stabilized with the increase in the nitrogen application level in different periods, and the highest nitrogen application level of maize yield should be between 270 and 360 kg/hm^2^. At the filling stage, canopy vegetation index of maize varieties with different nitrogen efficiencies was positively correlated with yield, dry matter weight, and leaf nitrogen content, especially GNDVI and GOSAVI on the leaf nitrogen content. It can be used as a means to predict its growth index.
